# Supplementary material for: SARS-CoV-2 N protein promotes NLRP3 inflammasome activation to induce hyperinflammation
Source: Nat Commun. 2021 Aug 2;12:4664. doi: 10.1038/s41467-021-25015-6 (PMC8329225; doi:10.1038/s41467-021-25015-6)
Supplement: Supplementary file 1 — Supplementary Information [file 41467_2021_25015_MOESM1_ESM.pdf]

SARS-CoV-2 N promotes the NLRP3 inflammasome activation to induce hyperinflammation

Supplementary Information

Supplementary Figures and Legends

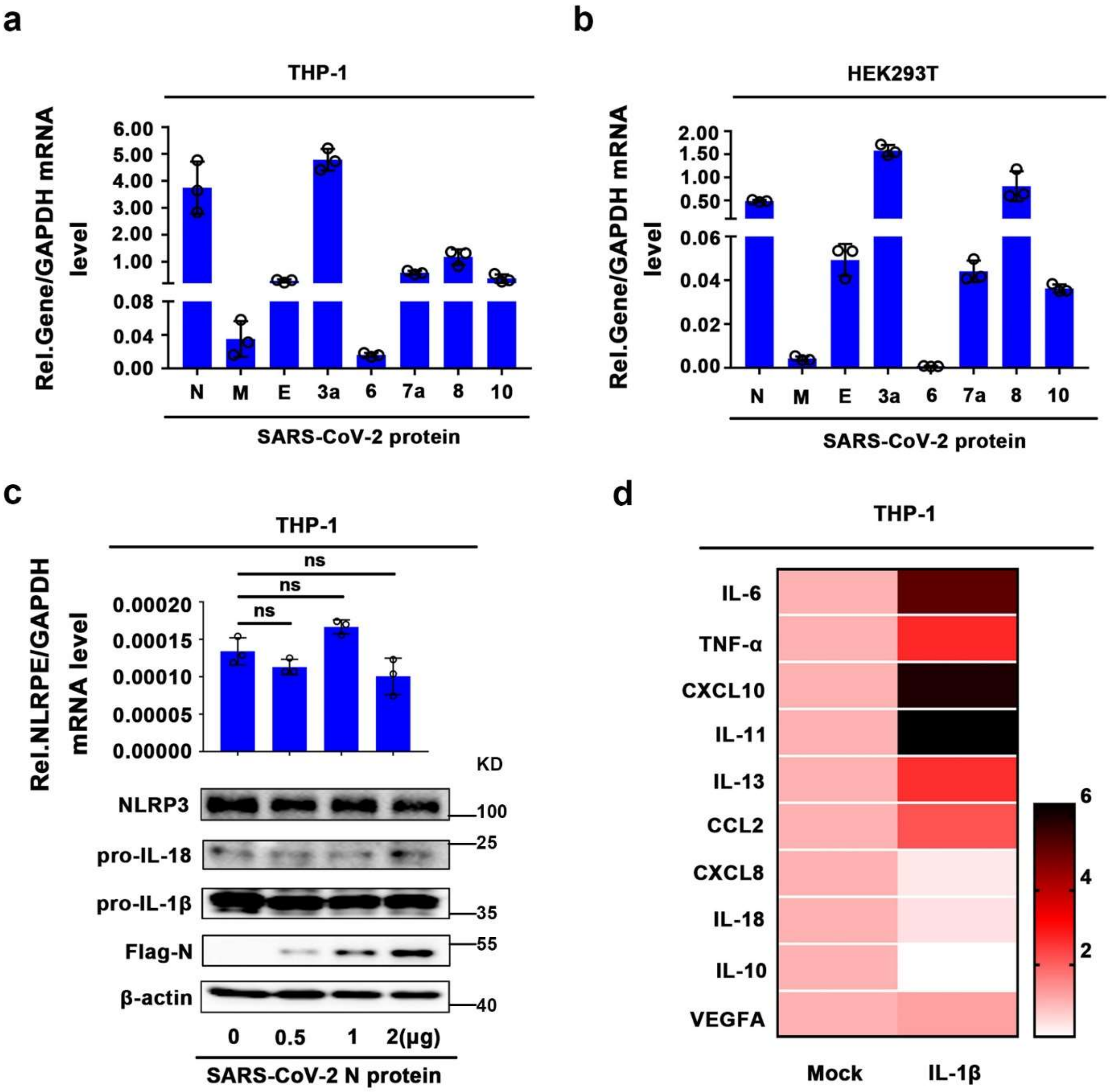

Supplementary Fig. 1. SARS-CoV-2 N protein induces proinflammatory responses. (**a**, **b**) PMA-differentiated THP-1 macrophages (a) or HEK293T cells (b) were transfected with plasmids encoding SARS-CoV-2 N, M, E, 3a, 6, 7a, 8, and 10 proteins at 2 μg for 48 h. The mRNA levels of indicated genes were quantified by qRT-PCR. Mock means untreated cells. (**c**) PMA-differentiated THP-1 macrophages were transfected with plasmids encoding SARS-CoV-2 N at different

concentrations for 48 h. NLRP3 mRNA was quantified by qRT-PCR. Proteins in the cell lysates were analyzed by immunoblotting. (**d**) PMA-differentiated THP-1 macrophages were stimulated with human IL-1β (100 ng/ml) for 2 h. The mRNA levels of indicated genes were quantified by qRT-PCR. Data are representative of three independent experiments and one representative is shown. Error bars indicate SD of technical triplicates, ns means not significant , two-tailed Student's t-test. Source data are provided as a Source Data file.

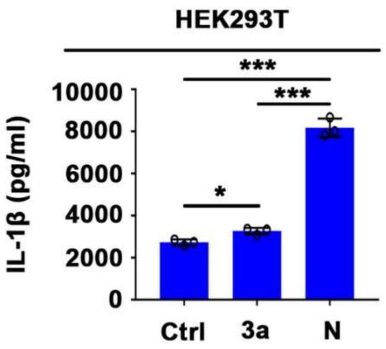

Supplementary Fig. 2. The effects of SARS-CoV-2 N and 3a proteins on IL-1β production. HEK293T cells were co-transfected with plasmids encoding NLRP3, ASC, pro-Casp1, and pro-IL-1β, and then transfected with plasmids encoding N protein or 3a protein for 48 h. The levels of IL-1β protein in the supernatants were analyzed by ELISA. Ctrl means transfected with empty plasmids. Data are representative of three independent experiments and one representative is shown. Error bars indicate SD of technical triplicates, $P \leq 0.05$ (*), $P \leq 0.01$ (**), $P \leq 0.001$ (***), two-tailed Student's t-test. Source data are provided as a Source Data file.

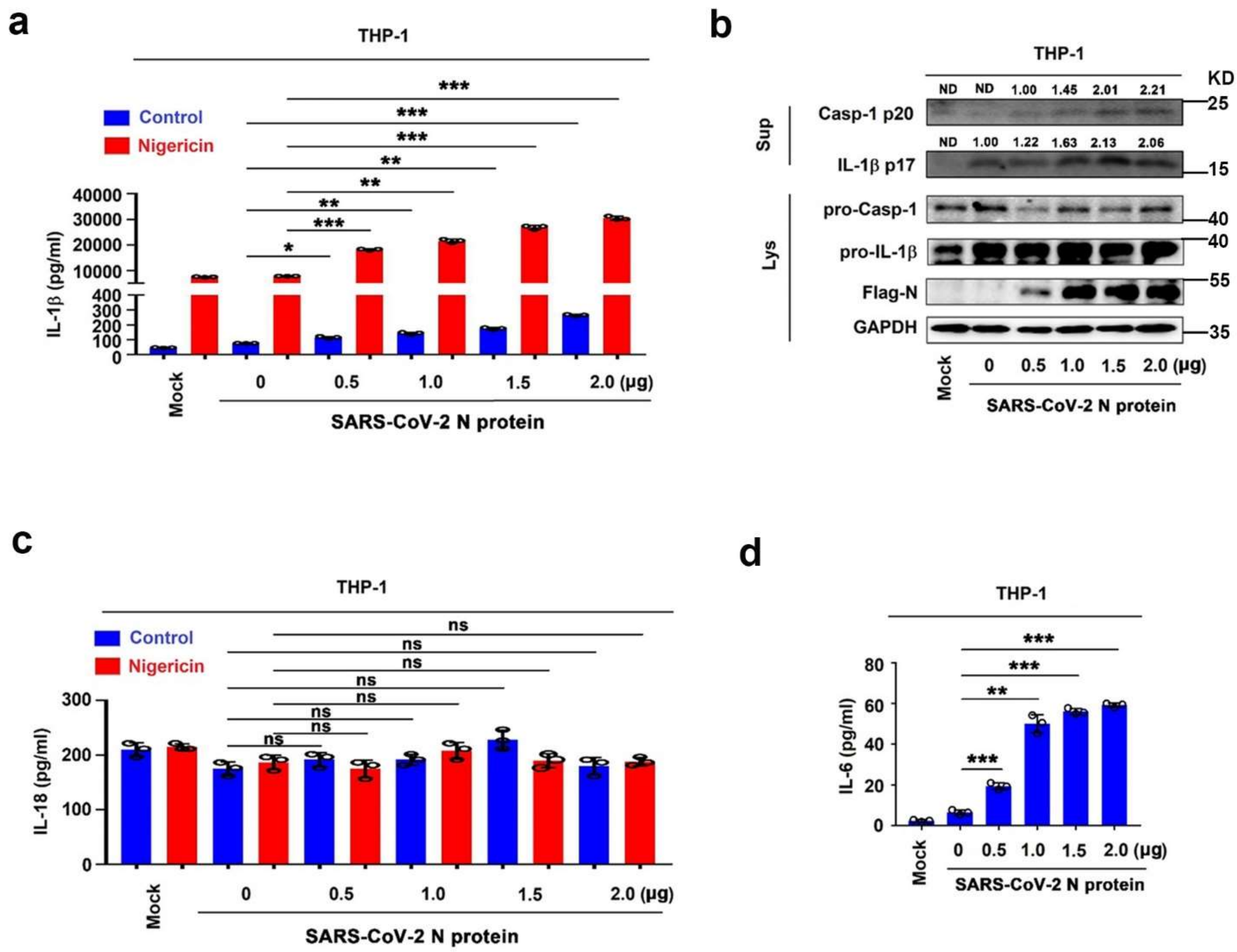

Supplementary Fig. 3. SARS-CoV-2 N induces IL-1β and IL-6 production. (**a**–**d**) PMA-differentiated THP-1 macrophages were transfected with plasmids encoding different concentrations of SARS-CoV-2 N for 48 h, then stimulated with 2 μM Nigericin or DMSO for 2 h. IL-1β (a), IL-18 (c) or IL-6 (d) in cell supernatants was measured by ELISA. Cell lysates were analyzed (b) by immunoblotting. Data are representative of three independent experiments and one representative is shown. Error bars indicate SD of technical triplicates, $P \leq 0.05$ (*), $P \leq 0.01$ (**), $P \leq 0.001$ (***), ns means not significant, two-tailed Student's t-test. Source data are provided as a Source Data file.

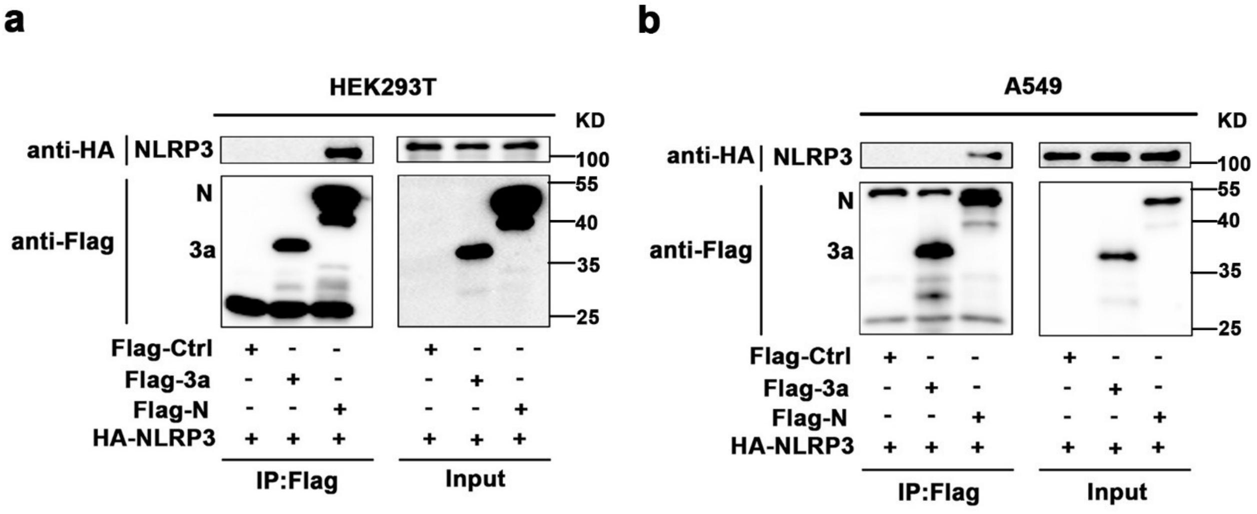

Supplementary Fig. 4. SARS-CoV-2 3a protein fails to interact with NLRP3. (**a**, **b**) HEK293T cells (a) or A549 cells (b) were co-transfected with HA-NLRP3 and Flag-N or Flag-3a. Proteins if the cell lysates were immunoprecipitated using anti-HA antibody and analyzed using anti-Flag and anti-HA antibody. Cell lysates (40 μg) was used as Input. Flag-Ctrl means transfected with empty plasmids. Data are representative of three independent experiments and one representative is shown. Source data are provided as a Source Data file.

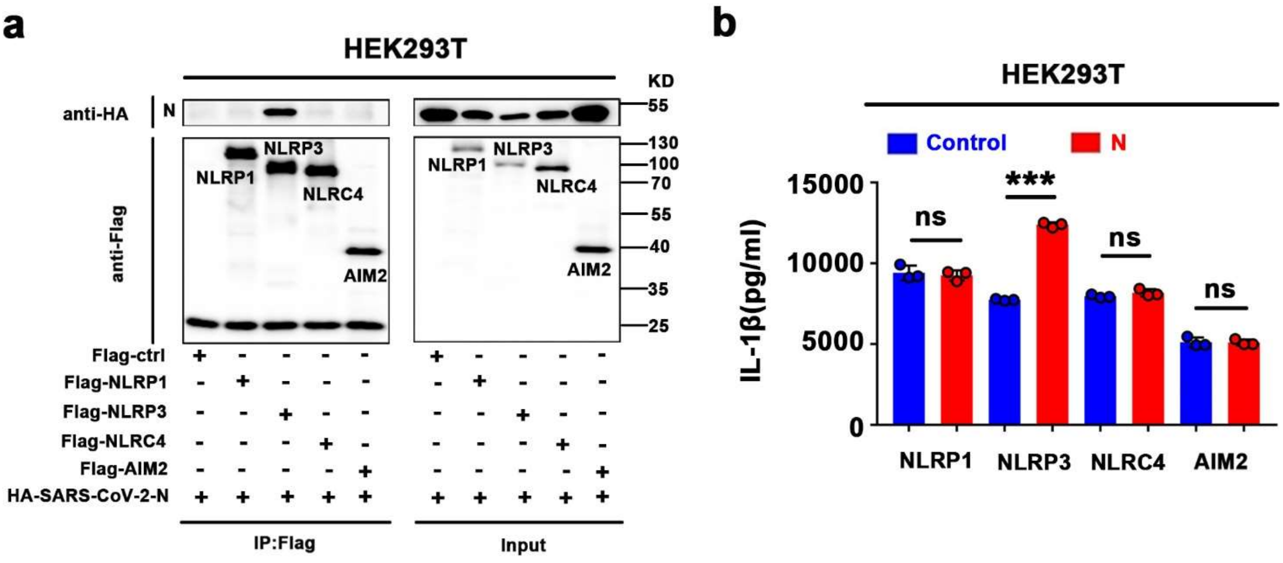

Supplementary Fig. 5. SARS-CoV-2 N protein interacts with NLRP3 and activates NLRP3 inflammasome. (**a**) HEK293T cells were co-transfected with HA-N and Flag-NLRP1, Flag-NLRP3, Flag-NLRC4, and Flag-AIM2 for 24 h. Proteins in the cell lysates were immunoprecipitated using anti-HA antibody and analyzed using anti-HA and anti-Flag antibody. Cell lysates (40 µg) were used as Inputs. (**b**) HEK293T cells were respectively co-transfected with plasmids encoding NLRP1inflammasome (NLRP1, pro-Casp1 and pro-IL-1β), NLRP3 inflammasome (NLRP3, ASC, pro-Casp1 and pro-IL-1β), NLRC4 inflammasome (NLRC4, ASC, pro-Casp1 and pro-IL-1β), AIM2 inflammasome (AIM2, ASC, pro-Casp1 and pro-IL-1β), and transfected with plasmids encoding SARS-CoV-2 N for 48 h. Supernatants were analyzed by ELISA for IL-1β. Flag-Ctrl (a) and Control (b) mean transfected with empty plasmids. Data are representative of three independent experiments and one representative is shown. Error bars indicate SD of technical triplicates, *P* ≤0.05 (*), *P* ≤0.01 (**), *P* ≤0.001 (***), ns means not significant, two-tailed Student's t-test. Source data are provided as a Source Data file.

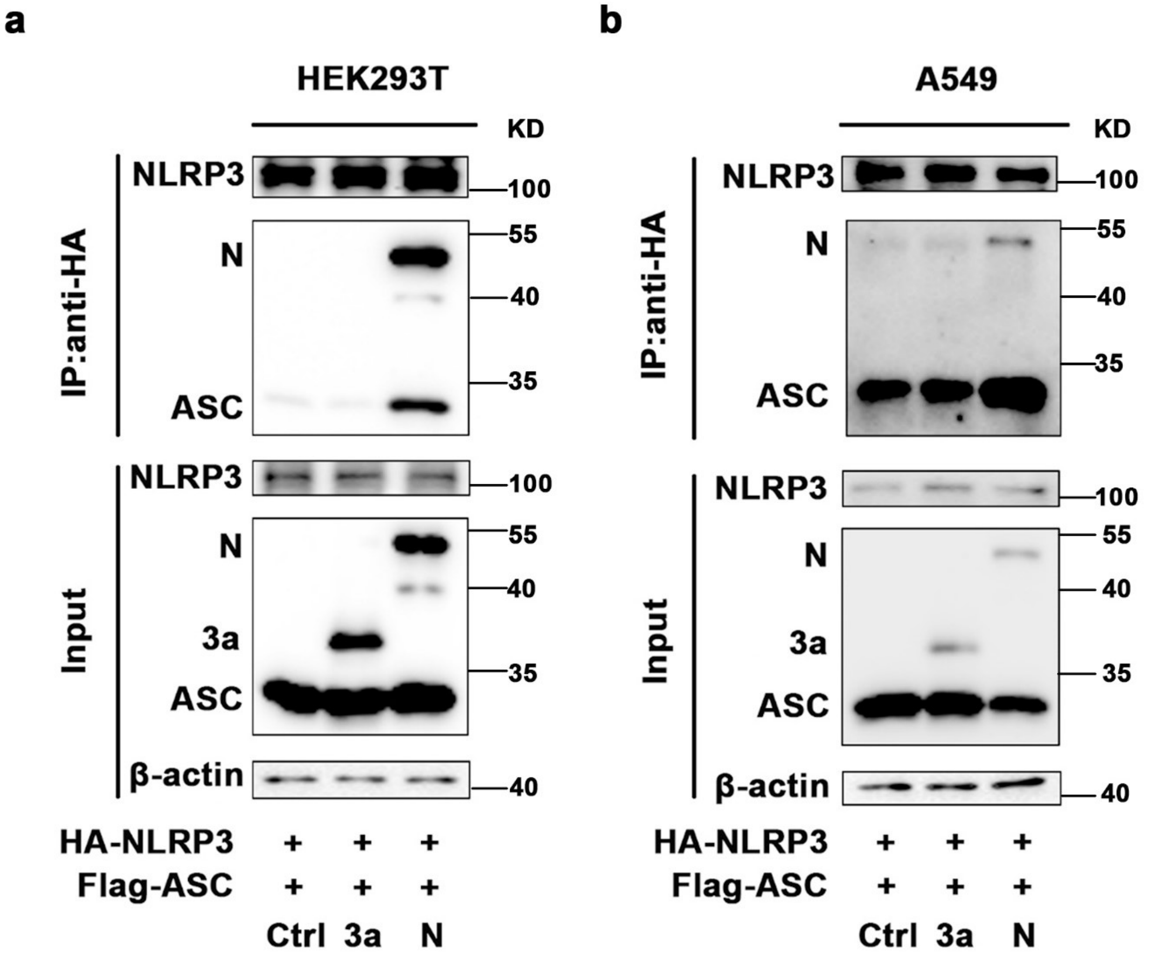

Supplementary Fig. 6. SARS-CoV-2 3a protein displays no effect on NLRP3-ASC interaction. (**a**, **b**) HEK293T cells (a) or A549 cells (b) were co-transfected with Flag-N or Flag-3a, HA-NLRP3 and Flag-ASC for 24 h. Proteins in the cell lysates were immunoprecipitated using anti-Flag antibody and analyzed using anti-HA and anti-Flag antibody. Cell lysates (40 μg) were used as Inputs. Ctrl means transfected with empty plasmids. Data are representative of three independent experiments and one representative is shown. Source data are provided as a Source Data file.

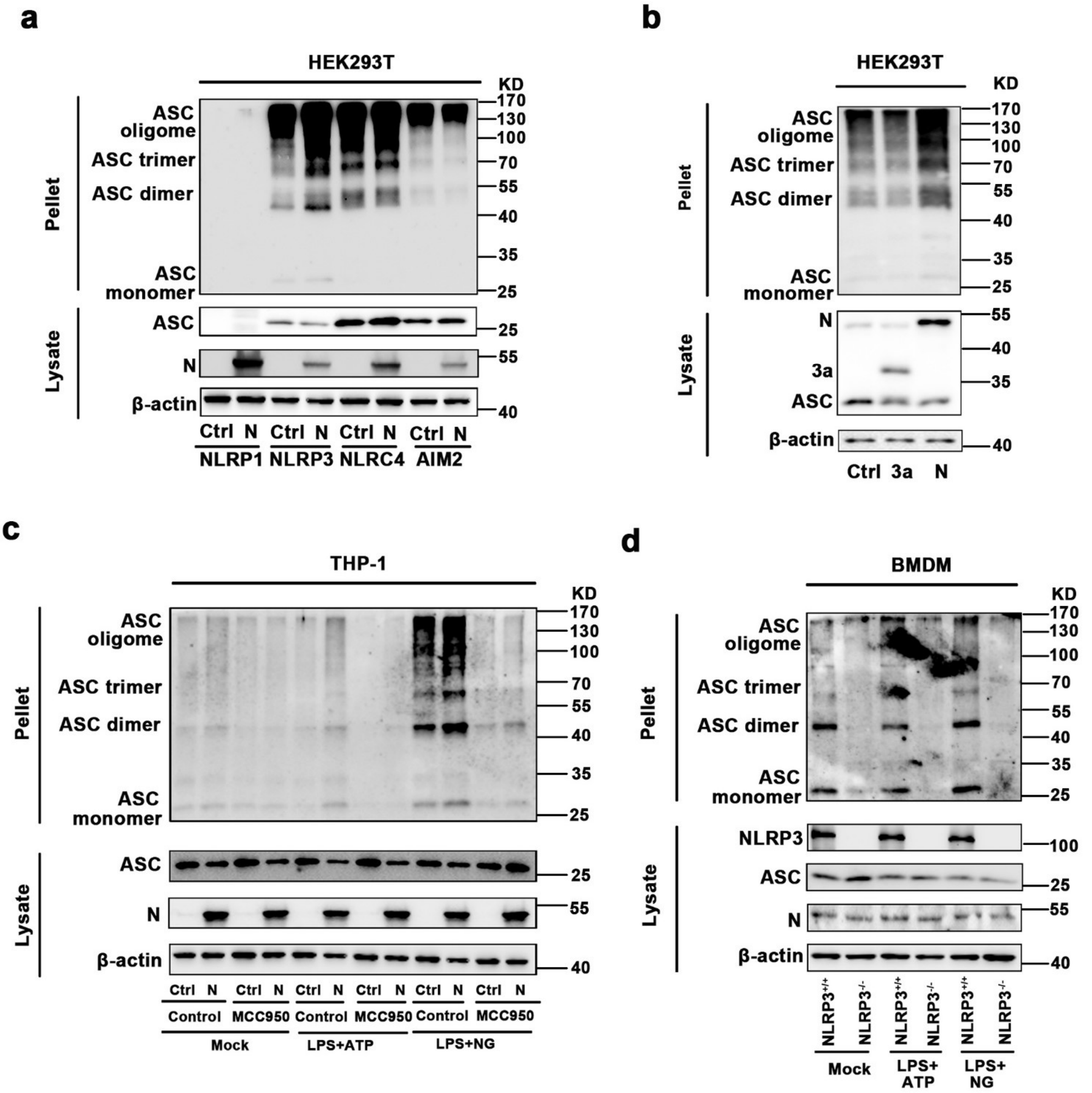

Supplementary Fig. 7. SARS-CoV-2 N protein promotes NLRP3-induced ASC oligomerization. (**a**) HEK293T cells were respectively co-transfected with plasmids encoding NLRP1inflammasome (NLRP1, pro-Casp1, and pro-IL-1β), NLRP3 inflammasome (NLRP3, ASC, pro-Casp1, and pro-IL-1β), NLRC4 inflammasome (NLRC4, ASC, pro-Casp1 and pro-IL-1β), AIM2 inflammasome (AIM2, ASC, pro-Casp1, and pro-IL-1β), and transfected with plasmids encoding SARS-CoV-2 N

for 48 h. ASC oligomerization was analyzed by immunoblotting. (**b**) HEK293T cells were co-transfected with plasmids encoding NLRP3, ASC, pro-Casp1, and pro-IL-1β, and then transfected with plasmids encoding SARS-CoV-2 N protein or 3a protein for 48 h. ASC oligomerization was analyzed by immunoblotting. (**c**) THP-1 macrophages were stably infected with Lentivirus-CT or Lentivirus-N, differentiated into macrophages. The cells were treated with MCC950 (0.01 μM) for 1 h, and then stimulated with LPS (1 μg/ml) plus ATP (2.5 mM) or LPS (1 μg/ml) plus Nigericin (2 μM). ASC oligomerization was analyzed by immunoblotting. (**d**) GM-CSF differentiated *NLRP3*$^{+/+}$ mice and *NLRP3*$^{-/-}$ mice BMDMs were infected with Lentivirus-N and then stimulated with LPS (1 μg/ml), LPS (1 μg/ml) plus ATP (2.5 mM), or LPS (1 μg/ml) plus Nigericin (2 μM). ASC oligomerization was analyzed by immunoblotting. Data are representative of three independent experiments and one representative is shown. Source data are provided as a Source Data file.

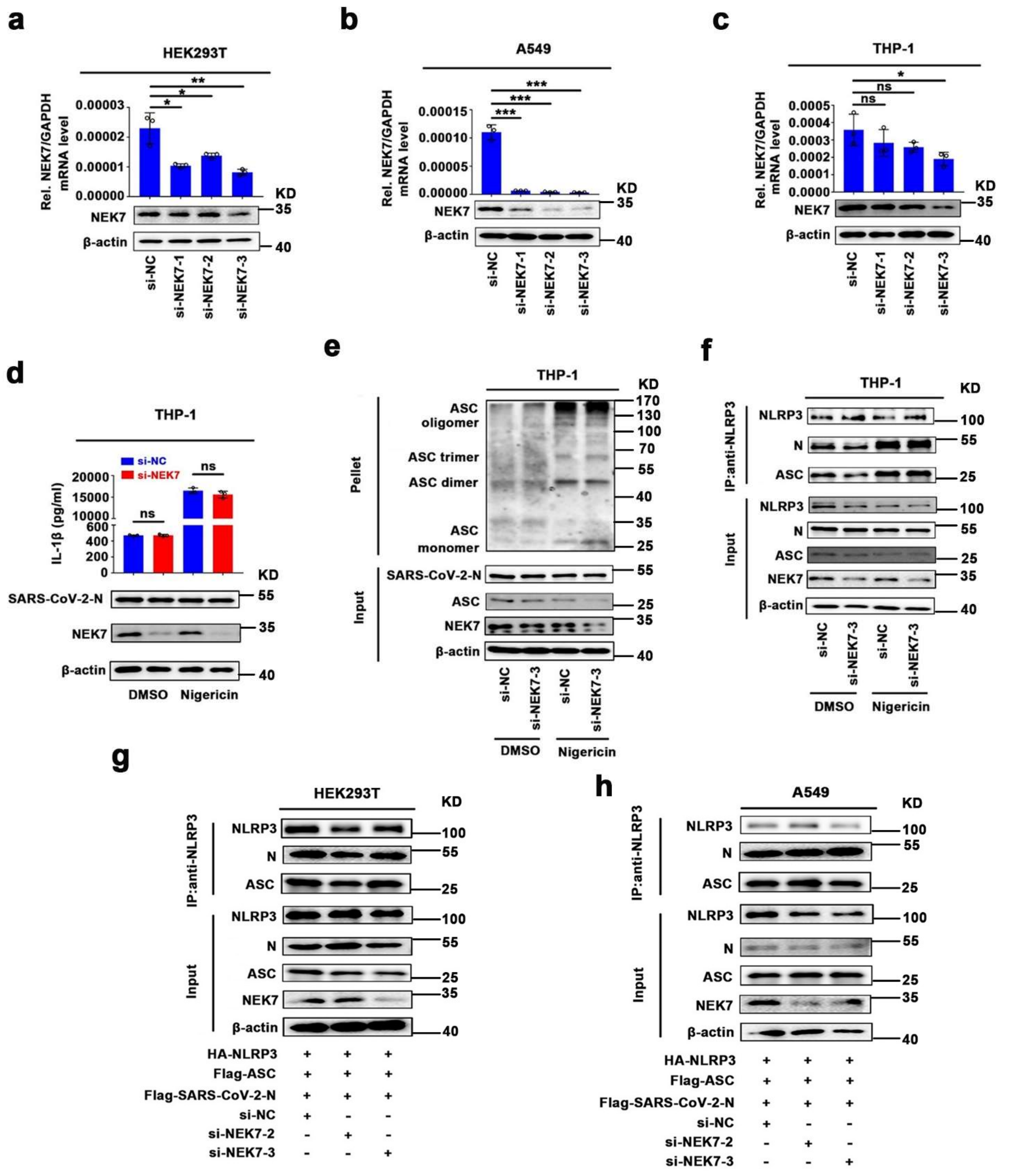

Supplementary Fig. 8. NEK7 is not required for N protein mediated inflammasome activation and NLRP3-ASC interaction. (**a**–**c**) HEK293T cells, A549 cells, or PMA-differentiated THP-1 macrophages were respectively transfected with si-NC, si-NEK7-1, si-NEK7-2, or si-NEK7-3 (50

nM) for 48 h. NEK7 mRNA was quantified by RT-PCR and NEK7 protein in lysates was analyzed by WB. (**d**–**f**) THP-1 macrophages were stably infected with Lentivirus-N were transfected with si-NC or si-NEK7-3 (50 nM) for 48 h, and stimulated with 2 μM Nigericin or DMSO for 2 h. IL-1β protein in the supernatants was analyzed by ELISA (d, top). Proteins in the cell lysates were analyzed by immunoblotting (d, bottom). ASC oligomerization was analyzed by immunoblotting (e). Cell lysates were immunoprecipitated using anti-NLRP3 antibody, and analyzed using anti-NLRP3, anti-ASC, anti-β-actin, and anti-N antibody (f). (**g**, **h**) HEK293T cells (g) or A549 cells (h) were transfected with si-NC, si-NEK7-2, or si-NEK7-3 (50 nM) for 24 h, and then co-transfected with Flag-SARS-CoV-2-N or 3a plus HA-NLRP3 and Flag-ASC for 24 h. Cell lysates were immunoprecipitated using anti-NLRP3 antibody, and analyzed using anti-NLRP3, anti-ASC, anti-β-actin, anti-NEK7 and anti-N antibody. Ctrl means transfected with empty plasmids. Data are representative of three independent experiments and one representative is shown. Error bars indicate SD of technical triplicates, $P \leq 0.05$ (*), $P \leq 0.01$ (**), $P \leq 0.001$ (***), ns means not significant, two-tailed Student's t-test. Source data are provided as a Source Data file.

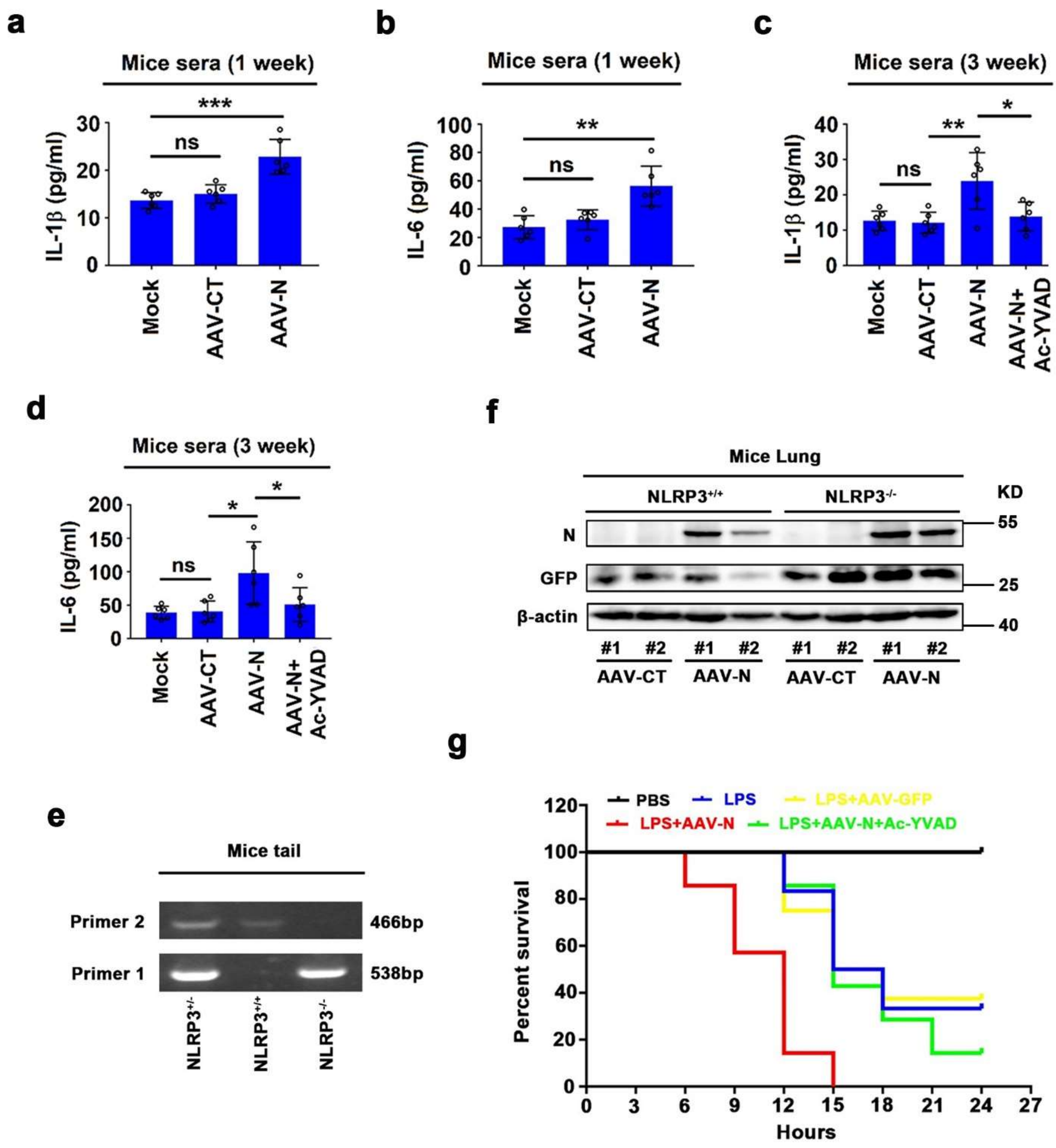

Supplementary Fig. 9. SARS-CoV-2 N protein induces mice lung injury via activating the NLRP3 inflammasome. (**a–d**) C57BL/6 genetic background mice were tail vein injection with 300 μl containing 5 × 10^{11} vg of AAV-Lung-EGFP (n=6) or AAV-Lung-N (n=16), after two weeks, treated with Ac-YVAD-cmk (8 mg/kg) by intraperitoneal injection for AAV-Lung-N (n=6) mice. Serum was collected at one week and three weeks for each group from the orbit. IL-1β (a and c) or

IL-6 (b and d) in the sera was measured by ELISA. Points represent the value of each serum samples. (**e**) The total genome DNA was extracted from the tail of *NLRP3*$^{+/+}$ mice, *NLRP3*$^{+/-}$ mice or *NLRP3*$^{-/-}$ mice. NLRP3 DNA was detected by specific primers. (**f**) *NLRP3*$^{+/+}$ C57BL/6 mice or *NLRP3*$^{-/-}$ C57BL/6 mice were tail vein injection with 300 μl containing 5×10^{11} vg of AAV-Lung-EGFP (n=4) or AAV-Lung-N (n=7). The indicated proteins were analyzed by WB. (**g**) After four weeks, pretreated with DMSO for AAV-Lung-N (n=7) mice or Ac-YVAD-cmk (8 mg/kg) for another AAV-Lung-N (n=7) mice. After 30 min, mock group (n=6), AAV-Lung-EGFP (n=7) group, AAV-Lung-N (n=7) group and another AAV-Lung-N (n=7) group were treated with LPS (30 mg/kg) by intraperitoneal injection. Another mock group (n=4) was intraperitoneal injected with PBS. Then the mice survival rates were evaluated every two hours posttreatment. Mock means inject the same dose of PBS as other groups (a–d). Data are representative of two independent experiments and one representative is shown. Error bars indicate SD of each serum samples (a–d). $P \leq 0.05$ (*), $P \leq 0.01$ (**), $P \leq 0.001$ (***), two-tailed Student's t-test (a–d). One-way ANOV analysis (g). Source data are provided as a Source Data file.

Supplementary Tables

Supplementary Table 1. qRT-PCR Primers used in this study.

**Name**	**Forward**	**Reverse**
Human IL-1β	5’-CACGATGCACCTGTACGATCA-3’	5’-GTTGCTCCATATCCTGTCCCT-3’
Human IL-6	5’-GTACATCCTCGACGGCATCTCA-3’	5’-GCACAGCTCTGGCTTGTTCCTC-3’
Human TNF-α	5’-TCTCGAACCCCGAGTGACA-3’	5’-GCCCGGCGGTTCA-3’
Human CXCL10	5’-ACTGTACGCTGTACCTGCAT-3’	5’-ACACGTGGACAAAATTGGCT-3’
Human GAPDH	5’-AAGGCTGTGGGCAAGG-3’	5’-TGGAGGAGTGGGTGTCG-3’
Human IL-11	5’-GAACTGTGTTTGCCGCCTGG-3’	5’-GTCTGGGGAAACTCGAGGGG-3’
Human IL-13	5’-CATGGCGCTTTTGTTGACCA-3’	5’-AGCTGTCAGGTTGATGCTCC-3’

Human CCL2	5’-TGCAATCAATGCCCCAGTCA-3’	5’-GGGTCAGCACAGATCTCCTT-3’
Human CXCL8	5’-CAGTTTTGCCAAGGAGTGCT-3’	5’-GTTTTCCTTGGGGTCCAGACA-3’
Human IL-18	5’-ACGCTTTACTTTATAGCTGAAGATG-3’	5’-GAGGCCGATTTCCTTGGTCA-3’
Human IL-10	5’-AGGCAACCTGCCTAACATGC-3’	5’-GTTCTCAGCTTGGGGCATCA-3’
Human VEGFA	5’-TGCGGATCAAACCTCACCAA-3’	5’-ACCAACGTACACGCTCCAG-3’
Mice IL-1β	5’-TGCCACCTTTTGACAGTGATG-3’	5’-TGTGCTGCTGCGAGATTTGA-3’
Mice IL-6	5’-AGACAAAGCCAGAGTCCTTCAGAGA-3’	5’-GCCACTCCTTCTGTGACTCCAGC-3’

Mice TNF-α	5’-ACGTGGAACTGGCAGAAGAG-3’	5’-CTCCTCCACTTGGTGGTTTG-3’
Mice CXCL10	5’-GTGCTGCCGTCATTTTCTGC-3’	5’-AAGCTTCCCTATGGCCCTCA-3’
Mice CCL2	5’-CCTGCTGCTACTCATTCACCA-3’	5’-ATTCCTTCTTGGGGTCAGCA-3’
Mice IL-11	5’-GAACTGTGTTTGTCGCCTGG-3’	5’-TGCTCGAGGGTCTGAAGAGA-3’
Mice IL-1β	5’-GAGGCCGATTTCCTTGGTCA-3’	5’-GAGGCCGATTTCCTTGGTCA-3’
Mice VEGFA	5’-CCCACGTCAGAGAGCAACAT-3’	5’-TGCGCTTTCGTTTTTGACCC-3’
Mice IL-7	5’-CGATGAATTGGACAAAATGACAGG-3’	5’-TGCGAGCAGCACGATTTAGA-3’

Mice CXCL8	5’-CCTGATGCTCCATGGGTGAA-3’	5’-ACAGAAGCTTCATTGCCGGT-3’
Mice IL-12A	5’-CTCAGTTTGGCCAGGGTCAT-3’	5’-GTCTTCAGCAGGTTTCGGGA-3’
Mice IL-18	5’-TCAGACAACTTTGGCCGACT-3’	5’-CAGTCTGGTCTGGGGTTCAC-3’
Mice IL-15	5’-GGGCTGTGTCAGTGTAGGTC-3’	5’-TGCAATTCCAGGAGAAAGCAG-3’
Mice IL-10	5’-GCATGGCCCAGAAATCAAGG-3’	5’-GAGAAATCGATGACAGCGCC-3’
Mice β-actin	5’-AGAGGGAAATCGTGCGTGAC-3’	5’-CAATAGTGATGACCTGGCCGT-3’
Human NLRP3	5-AAGGGCCATGGACTATTTCC-3’	5’-GACTCCACCCGATGACAGTT-3’

Human NEK7	5-CAGATGCTGGCGACCTATCC-3’	5’-TCCAATGCACTGCAAAGCTG-3’
SARS-CoV-2 N	5-ACCCGCAATCCTGCTAACAA-3’	5-ACGAGAAGAGGCTTGACTGC-3’
SARS-CoV-2 M	5-GTGCCACTCCATGGCACTAT-3’	5’-TCCTTGATGTCACAGCGTCC-3’
SARS-CoV-2 E	5-TTCGTTTCGGAAGAGACAGGT-3’	5-GCGCAGTAAGGATGGCTAGT-3’
SARS-CoV-2 3a	5-GCTTTGCTGGAAATGCCGTT-3’	5’-GGACTTGTTGTGCCATCACC-3’
SARS-CoV-2 6	5-TCATCTCGTTGACTTTCAGGTT-3’	5-TCTCCATTGGTTGCTCTTCATCT-3’
SARS-CoV-2 7a	5-TGGCACTGATAACACTCGCT-3’	5-CCGTCAGGACAAGCAAAAGC-3’
SARS-CoV-2 8	5-GAATTGTGCGTGGATGAGGC-3’	5-ACCCAATTTAGGTTCCTGGCA-3’

SARS-CoV-2 10	5’-AACGTTTTCGCTTTTCCGTT-3’	5-TCTACTTGTGCTATGTAGTTACGA-3’

Supplementary Table 2. Clone Primers used in this study.

Name	Forward	Reverse
N1	5’-GCGGATCCATGTCTGATAATGGACCCCAA-3’	5’-GCTCTAGATTAAATGCGCGACATTCCGA-3’
N2	5’-GCGGATCCATGTCTGATAATGGACCCCAA-3’	5’-GCTCTAGATTATTGCCGAGGCTTCTTAG-3’
N3	5’-GCGGATCCATGTCTGATAATGGACCCCAA-3’	5’-GCTCTAGATTAACTGCCGCCTCTGCTC-3’
N4	5’-GCGGATCCATGTCTGATAATGGACCCCAA-3’	5’-CGTCTAGATTAAGCTCTTCGGTAGTAGC-3’
N5	5’-GCGGATCCATGACCAGACGAATTCG-3’	5’-GCTCTAGATTAGGCCTGAGTTGAGTC-3’

N6	5’-GCGGATCCATGCAAGCCTCTTCTCGT-3’	5’-GCTCTAGATTAGGCCTGAGTTGAGTC-3’
N7	5’-GCGGATCCATGAAACGTACTGCCAC-3’	5’-GCTCTAGATTAGGCCTGAGTTGAGTC-3’
N8	5’-GCGGATCCATGGACAAAGATCCAAA-3’	5’-GCTCTAGATTAGGCCTGAGTTGAGTC-3’
