# Supplementary material for: SARS-CoV-2 N protein promotes NLRP3 inflammasome activation to induce hyperinflammation
Source: Nat Commun. 2021 Aug 2;12:4664. doi: 10.1038/s41467-021-25015-6 (PMC8329225; doi:10.1038/s41467-021-25015-6)
Supplement: Supplementary file 3 — Description of Additional Supplementary Files [file 41467_2021_25015_MOESM3_ESM.pdf]

Description of Additional Supplementary Files

File Name: Supplementary Movie 1
Description: N protein and NLRP3 protein were co-localized in the cytoplasm of A549 cells.

File Name: Supplementary Movie 2
Description: N protein and ASC protein were not co-localized in the cytoplasm of HEK293T cells.

File Name: Supplementary Movie 3
Description: N protein and ASC protein were not co-localized in the cytoplasm of A549 cells.

File Name: Supplementary Movie 4
Description: N, ASC, and NLRP3 were obviously co-localized to form “sphere-like” structures in HEK293T cells.

File Name: Supplementary Movie 5
Description: N, ASC, and NLRP3 were obviously co-localized to form “sphere-like” structures in A549 cells.
