# Supplementary material for: SARS-CoV-2 N protein promotes NLRP3 inflammasome activation to induce hyperinflammation
Source: Nat Commun. 2021 Aug 2;12:4664. doi: 10.1038/s41467-021-25015-6 (PMC8329225; doi:10.1038/s41467-021-25015-6)
Supplement: Supplementary file 9 — Reporting Summary [file 41467_2021_25015_MOESM9_ESM.pdf]

nature research

Corresponding author(s): Geng Li, Yongkui Li, and Jianguo Wu

Last updated by author(s): Jul 10, 2021

Reporting Summary

Nature Research wishes to improve the reproducibility of the work that we publish. This form provides structure for consistency and transparency in reporting. For further information on Nature Research policies, see our Editorial Policies and the Editorial Policy Checklist.

Statistics

For all statistical analyses, confirm that the following items are present in the figure legend, table legend, main text, or Methods section.

n/a	Confirmed	
☐	☒	The exact sample size (*n*) for each experimental group/condition, given as a discrete number and unit of measurement
☐	☒	A statement on whether measurements were taken from distinct samples or whether the same sample was measured repeatedly
☐	☒	The statistical test(s) used AND whether they are one- or two-sided<br>*Only common tests should be described solely by name; describe more complex techniques in the Methods section.*
☐	☒	A description of all covariates tested
☐	☒	A description of any assumptions or corrections, such as tests of normality and adjustment for multiple comparisons
☐	☒	A full description of the statistical parameters including central tendency (e.g. means) or other basic estimates (e.g. regression coefficient) AND variation (e.g. standard deviation) or associated estimates of uncertainty (e.g. confidence intervals)
☐	☒	For null hypothesis testing, the test statistic (e.g. F, t, r) with confidence intervals, effect sizes, degrees of freedom and P value noted<br>*Give P values as exact values whenever suitable.*
☐	☒	For Bayesian analysis, information on the choice of priors and Markov chain Monte Carlo settings
☐	☒	For hierarchical and complex designs, identification of the appropriate level for tests and full reporting of outcomes
☒	☐	Estimates of effect sizes (e.g. Cohen's *d*, Pearson's *r*), indicating how they were calculated

Our web collection on statistics for biologists contains articles on many of the points above.

Software and code

Policy information about availability of computer code

Data collection	There are no software was used for data collection
Data analysis	GraphPad Prism 7 and Image J (1.48v) were used for Data analysis

For manuscripts utilizing custom algorithms or software that are central to the research but not yet described in published literature, software must be made available to editors and reviewers. We strongly encourage code deposition in a community repository (e.g. GitHub). See the Nature Research guidelines for submitting code & software for further information.

Data

Policy information about availability of data

All manuscripts must include a data availability statement. This statement should provide the following information, where applicable:

- Accession codes, unique identifiers, or web links for publicly available datasets
- A list of figures that have associated raw data
- A description of any restrictions on data availability

Data availability statement has been added in our manuscript. Data availability: GSE155106, RNA-Seq data from GEO database (https://www.ncbi.nlm.nih.gov/geo/query/acc.cgi?acc=GSE155106). All other data are included in the Article and Supplemental Information or available from the authors upon reasonable requests. Source data are provided with this paper.

Field-specific reporting

Please select the one below that is the best fit for your research. If you are not sure, read the appropriate sections before making your selection.

☒ Life sciences ☐ Behavioural & social sciences ☐ Ecological, evolutionary & environmental sciences

For a reference copy of the document with all sections, see nature.com/documents/nr-reporting-summary-flat.pdf

Life sciences study design

All studies must disclose on these points even when the disclosure is negative.

Sample size	For cell studies, the control samples or experimental samples both consisting of three independent samples. For animal studies, the control samples or experimental samples both consisting of eight mices.
Data exclusions	There are not data were excluded from the analyses unless the recording quality was poor due to the technical fators which make data interpretation difficult. For example: Man-made experimental errors or Random contamination of experimental samples
Replication	For cell experiments, the dates were representative of three independent experiments, For animal experiments, the dates were representative of two independent experiments, and all attempts at replication were successful.
Randomization	For cell experiments, Cells from the mother flask were randomly placed in different cell dishes, each experiment were representative of three independent experiments.
Blinding	Blinding were not involved in this study

Reporting for specific materials, systems and methods

We require information from authors about some types of materials, experimental systems and methods used in many studies. Here, indicate whether each material, system or method listed is relevant to your study. If you are not sure if a list item applies to your research, read the appropriate section before selecting a response.

Materials & experimental systems

n/a	Involved in the study	
☐	☒	Antibodies
☐	☒	Eukaryotic cell lines
☒	☐	Palaeontology and archaeology
☐	☒	Animals and other organisms
☒	☐	Human research participants
☒	☐	Clinical data
☒	☐	Dual use research of concern

Methods

n/a	Involved in the study	
☒	☐	ChIP-seq
☒	☐	Flow cytometry
☒	☐	MRI-based neuroimaging

Antibodies

Antibodies used	Anti-NLRP3 (D4d8T, 1:1000), anti-Caspase-1 (D7F10, 1:1000), and anti-IL-1β (D3U3E, 1:1000) antibodies were purchased from Cell Signaling Technology. Anti-NLRP3 (AG-20B-0006, 1:200) were purchased from AdipoGen Life Science. Anti-SARS-CoV-2-N (A20021, 1:1000), anti-NEK7 (A19816, 1:1000), and anti-β-actin (AC026, 1:2000) antibody was purchased from ABclonal. Anti-Flag (F3165, 1:1000), anti-HA (H6908, 1:2000), and anti-GAPDH (G8759, 1:2000) were purchased from Sigma. Anti-ASC (sc-271054, 1:500) was purchased from Santa Cruz Biotechnology. Rabbit IgG (PA1-28573, 1:5000) and Mouse IgG (31464, 1:5000) were purchased from Invitrogen. Anti-mouse/rabbit IgG Dylight 649 (A23620, 1:200), anti-mouse/rabbit IgG Dylight cy3 (A22210, 1:200), and anti-mouse/rabbit IgG FITC (A22110, 1:200) were purchased from Abbkine .
Validation	All the primary antibody were useful. NLRP3 (D4D8T) Rabbit mAb #15101 (https://www.cellsignal.cn/products/primary-antibodies/nlrp3-d4d8t-rabbit-mab/15101?_=1625891099414&Ntt=d4d8t&tahead=true) relevant citations: 133. Caspase-1 (D7F10) Rabbit mAb #3866(https://www.cellsignal.cn/products/primary-antibodies/caspase-1-d7f10-rabbit-mab/3866?_=1625891113267&Ntt=d7f10&tahead=true)relevant citations: 75. IL-1β (D3U3E) Rabbit mAb #12703(https://www.cellsignal.cn/products/primary-antibodies/il-1b-d3u3e-rabbit-mab/12703?_=1625891179181&Ntt=d3u3e&tahead=true)relevant citations: 82.anti-NLRP3/NALP3 (mouse), mAb (Cryo-1)(https://adipogen.com/ag-20b-0006-anti-nlrp3-nalp3-mouse-mab-cryo-1.html). SARS-CoV-2 N Protein Rabbit mAb (A20021) (https://abclonal.com.cn/catalog/A20021). relevant citations: 1. NEK7 Rabbit mAb (A19816) (https://abclonal.com.cn/catalog/A19816). β-Actin Rabbit mAb (High Dilution) (AC026)(https://abclonal.com.cn/catalog/AC026).relevant citations: 389. Anti-Flag (F3165, 1:1000) (https://www.sigmaaldrich.cn/CN/zh/product/sigma/f3165?context=product).anti-HA (H6908, 1:2000)(https://www.sigmaaldrich.cn/CN/zh/product/sigma/h6908?context=product).anti-GAPDH (G8759, 1:2000)(https://www.sigmaaldrich.cn/CN/zh/product/sigma/g8759?context=product).Anti-ASC/TMS1/PYCARD Antibody (F-9): sc-271054.(https://www.scbt.com/p/asc-antibody-f-9).relevant citations: 78.Rabbit anti-Rat IgG (H+L) Secondary Antibody, HRP(https://www.thermofisher.cn/cn/zh/antibody/product/Rabbit-anti-Rat-IgG-H-L-Secondary-Antibody-Polyclonal/

PA1-28573).Mouse anti-Rabbit IgG (H+L) Cross-Adsorbed Secondary Antibody, HRP(https://www.thermofisher.cn/cn/zh/antibody/product/Mouse-anti-Rabbit-IgG-H-L-Cross-Adsorbed-Secondary-Antibody-Polyclonal/31464).Anti-mouse/rabbit IgG Dylight 649 (A23620)(http://www.abbkine.cn/product/a23620).relevant citations: 13. anti-mouse/rabbit IgG Dylight cy3 (A22210).http://www.abbkine.cn/product/a22210.relevant citations: 9. and anti-mouse/rabbit IgG FITC (A22110)(http://www.abbkine.cn/product/a22110).relevant citations: 10.

Eukaryotic cell lines

Policy information about cell lines

Cell line source(s)	THP-1 cell(Cat#:TIB-2-2) ,HEK293T cell(Cat#:CRL-3216) ,A549 cell (Cat#:CCL-185) were purchased from ATCC
Authentication	STR profiling was used for authenticate each cell line
Mycoplasma contamination	All the cell lines were tested negative for mycoplasma contamination.
Commonly misidentified lines (See ICLAC register)	There are no commonly misidentified cell lines were used in the study.

Animals and other organisms

Policy information about studies involving animals; ARRIVE guidelines recommended for reporting animal research

Laboratory animals	NLRP3+/+ C57BL/6 or NLRP3-/- C57BL/6 genetic background mice were bred and maintained under specific pathogen-free conditions, 6-week-old mice, equal numbers of female and male mice were used and All the mice were bred and maintained under specific pathogen-free conditions. Lighting time can be set at 8:00-20:00, automatic control, The temperature is 22 ℃, Full use of independent ventilation system (IVC)
Wild animals	This study does not involve any wild animals
Field-collected samples	this study does not involve any field-collected samples.
Ethics oversight	All animal studies were performed in accordance with the principles described by the Animal Welfare Act and the National Institutes of Health Guidelines for the care and use of laboratory animals in biomedical research. All procedures involving mice and experimental protocols were approved by Institute of laboratory animal science, Jinan University. The animal ethic committee number is 20200828-09. All the mice that need to be sacrificed were used the method of euthanasia.

Note that full information on the approval of the study protocol must also be provided in the manuscript.
